# Conventional DNA Extraction Followed by Real-Time PCR Had Higher Sensitivity for Detection of *Mycobacterium Tuberculosis* in Clinical Samples Compared to Standard Methods

**DOI:** 10.4269/ajtmh.24-0797

**Published:** 2025-04-01

**Authors:** Angel Sebastian Rodriguez-Pazmiño, Bernardo Castro-Rodríguez, Greta Esther Cardenas-Franco, Greta Franco-Sotomayor, Elsy Carvajal, Joselyn Calderon, Darwin Santiago Paredes, Heidy Buenaño-Morales, Manuel González, Lina Macero, Rita García, Solon Alberto Orlando, Miguel Angel Garcia-Bereguiain

**Affiliations:** ^1^ One Health Research Group, Universidad de Las Américas, Quito, Ecuador;; ^2^ Instituto Nacional de Salud Pública e Investigación, Guayaquil, Ecuador;; ^3^ Universidad Católica Santiago de Guayaquil, Guayaquil, Ecuador;; ^4^ Universidad Ecotec, Guayaquil, Ecuador;; ^5^ Hospital de Infectología “Dr. José Daniel Rodríguez Maridueña,” Ministerio de Salud Pública, Guayaquil, Ecuador;; ^6^ Universidad Espíritu Santo, Guayaquil, Ecuador

## Abstract

The *Mycobacterium tuberculosis complex* (MTBC), the causative agent of tuberculosis (TB), remains a significant global health challenge, with an estimated 10.8 million cases diagnosed and 1.25 million deaths in 2023, according to the WHO. In this context, enhancing TB case detection using more sensitive diagnostic methods is essential. Here, we compared the performance of two commercial real-time polymerase chain reaction (PCR) kits with the reference standards of smear microscopy, culture, and Gene Xpert. By analyzing 402 clinical specimens, we found that conventional DNA extraction, followed by real-time PCR, using either of the two commercial kits provided the highest sensitivity for detecting MTBC. Positivity values of 48.98–60.07% and 50.34–53.24% were obtained with the commercial kits “VIASURE MTBC + non tuberculous mycobacterias (NTM) Real-Time PCR Detection Kit” (Certest, Spain) and “ANYPLEX™ MTB/NTM Real-Time Detection Kit” (Seegene, South Korea), respectively. In contrast, the reference standards yielded positivity values of 14.75% (smear microscopy), 32.65% (culture), and 28.95% (GeneXpert assay). These alternative methods should be considered as valuable tools to strengthen TB control and prevention strategy.

The *Mycobacterium tuberculosis complex* (MTBC), the causative agent of tuberculosis (TB), represents the most clinically significant group of mycobacteria. According to the WHO’s 2024 Global TB Report, around 10.8 million new TB cases and 1.25 million deaths occurred in 2023 worldwide.^1^ After being overtaken by coronavirus disease 2019 for 3 years, TB is likely back to being the world’s leading infectious agent-related cause of death. In addition, it is the primary cause of death from antimicrobial resistance and the leading cause of death for HIV-positive individuals.^2^ Continuous improvement of diagnostics is a key part of WHO’s “End TB Strategy,” which aims for the majority eradication of the disease by 2030.^3^ In this regard, clinical and bacteriological criteria have been traditionally used to diagnose a patient suspected of having TB in middle-to-high burden settings, usually low- and middle-income countries.^4^ The laboratory diagnostic algorithms usually include a combination of smear microscopy, culture, and GeneXpert assay. However, there has been a trend to switch to exclusively using the GeneXpert assay for TB control and prevention in recent years in the Americas. This is the case of Ecuador, which has promoted the exclusive use of GeneXpert within the national surveillance program since 2024.

In this study, we present the results of our experience carrying out a comparative analysis of the performance of smear microscopy, culture, GeneXpert assay (conventional tests recommended by WHO), and two commercial real-time polymerase chain reaction (PCR) kits for detecting MTBC available for clinical use in Ecuador. The smear microscopy procedure was performed according to the Association of Public Health Laboratories Essentials,^5^ the Kudoh-Owaga swab method was used for culture based on the methodology described in Franco-Sotomayor et al.,^6^ and the procedure in GeneXpert assay was done according to manufacturer’s instructions.^7^ The two commercial real-time PCR kits included were: 1) “VIASURE MTBC + non tuberculous mycobacterias (NTM) Real-Time PCR Detection Kit” (Certest, Spain) (from now we referred to this kit as “VIASURE kit”), 2) “ANYPLEX™ MTB/NTM Real-Time Detection Kit” (Seegene, South Korea) (from now we referred to this kit as “ANYPLEX kit”). Both commercial real-time PCR kits are designed to simultaneously detect and discriminate bacteria of the genus *Mycobacterium*, either MTBC or non-tuberculous mycobacteria, in clinical samples from patients with signs and symptoms of TB with a single multiplex PCR reaction.^8^^,^^9^

A total of 402 clinical samples from patients with signs and symptoms of TB (recruited for TB screening according to clinical algorithm for the national TB surveillance program in Ecuador) were included in this study, approved by the Institutional Review Board of “Hospital Luiz Vernaza” from Guayaquil (Ecuador) with code 9-EO-CEISH-HLV-24. From those 402 clinical specimens, there were smear microscopy results for 278 samples, MTBC culture results for 147 samples, and GeneXpert assay results for 228 samples (Table 1; Figure 1). The 402 clinical specimens were preserved at –80°C until they were subjected to DNA extraction using a commercial column-based DNA extraction kit and then tested with the two real-time PCR kits for MTBC detection following the instructions manual for each commercial brand.

**Table 1:** The MTBC detection positivity rates for the different diagnosis tests included in this study

Group	Test	Total Valid Samples	Total Positives (%)	CIs (95%)
1	Smear microscopy	278	14.75 (*n* = 41)	10.58–18.92
	VIASURE real-time PCR		60.07 (*n* = 167)	54.31–65.83
	Anyplex real-time PCR		53.24 (*n* = 148)	47.37–59.11
2	Culture	147	32.65 (*n* = 48)	25.07–40.23
	VIASURE real-time PCR		48.98 (*n* = 72)	40.90–57.06
	Anyplex real-time PCR		50.34 (*n* = 74)	42.26–58.42
3	GeneXpert assay	228	28.95 (*n* = 66)	23.06–34.84
	VIASURE real-time PCR		57.02 (*n* = 130)	50.59–63.45
	Anyplex real-time PCR		52.63 (*n* = 120)	46.15–59.11

MTBC = *Mycobacterium tuberculosis complex*; PCR = polymerase chain reaction. The total number of samples was 402, of which 278 were used for group 1, 147 for group 2, and 228 for group 3. Samples were divided according to information available for each reference-standard method: smear microscopy, culture, and GeneXpert assay. For each group, the comparison with commercial real-time polymerase chain reaction kits is shown (% of positive samples and 95% CIs are shown).

**Figure 1.**
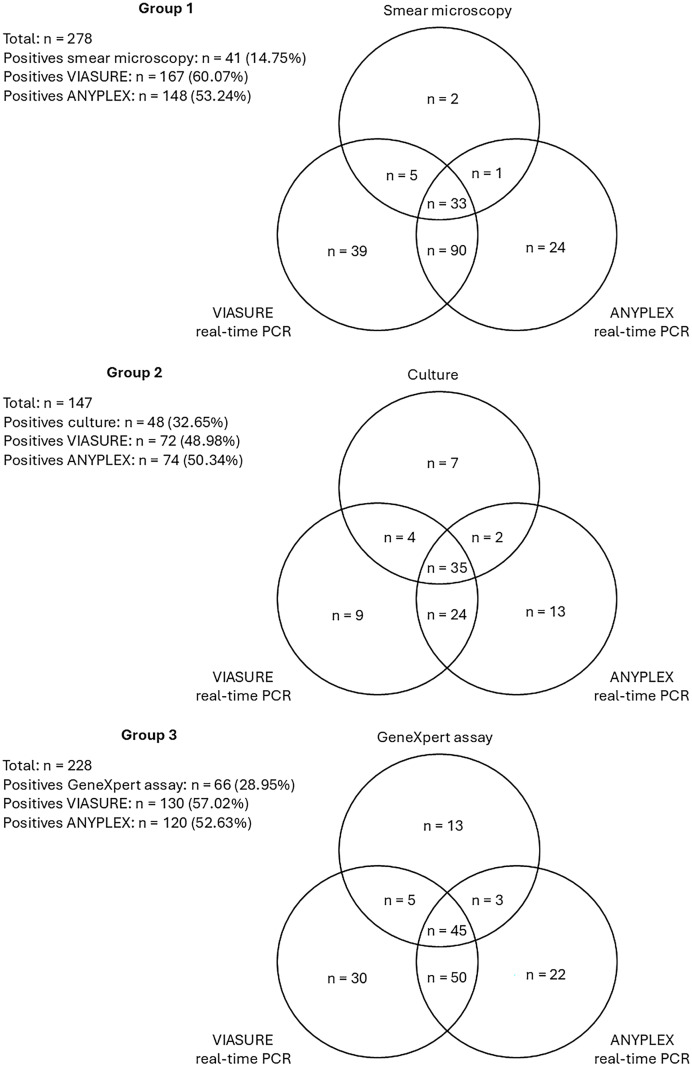
Venn diagrams on the comparison of the positive results of the three groups of samples detailed in Table 1. Each circle includes only positives samples for each method tested.

The results of smear microscopy, MTBC GeneXpert assay, and commercial real-time PCR kits are presented in Table 1 and Figure 1. The positivity rates for MTBC detection for the different diagnosis tests included in this study are shown for clinical samples divided into three groups according to information available for each reference standard: smear microscopy, culture, and GeneXpert assay (Table 1). Commercial PCR kits consistently show statistically significantly higher percentages of positive results in the three groups than conventional tests (*P*-value 0.05 for Kruskal-Wallis and Dunn statistical tests; these analyses were performed in R version 4.4.1). The CIs were also calculated for the positivity percentages (Table 1). Although the MTBC-positivity rate for smear microscopy was 14.75%, the rates for the VIASURE and ANYPLEX kits were 60.07% and 53.24%, respectively. Although the MTBC-positivity rate for MTBC culture was 32.65%, the rates for the VIASURE and ANYPLEX kits were 48.98% and 50.44%, respectively. Finally, although the MTBC-positivity rate for the GeneXpert assay was 28.85%, the rates for the VIASURE and ANYPLEX kits were 57.02% and 52.62%, respectively.

Overall, our results clearly show that a simple DNA extraction with commercial column-based kits, followed by a real-time PCR with a commercial kit, is a procedure with higher sensitivity for detecting MTBC directly for clinical specimens than the reference standards endorsed by WHO like smear microscopy, culture, or GeneXpert assay (Figure 1). This suggests that these techniques could potentially identify mycobacterial DNA more efficiently even in samples with low bacillary load. On the other hand, as it is well known, smear microscopy has the lowest positivity (14.75% in our study) among the reference standard methods. Although GeneXpert assay and culture show higher positivity rates compared with smear microscopy as expected, they still fall short compared with commercial PCR kits. Moreover, the performance of both commercial PCR kits was similar across all three groups (Figure 1), suggesting that both assays have comparable sensitivities within the evaluated set of samples.

This study was conducted in collaboration with the National Reference Center for Mycobacteria from the National Institute of Public Health from Ecuador in Guayaquil, as well as with hospitals within Ecuador’s national network for TB surveillance and control. Traditionally, TB diagnostic guidelines in Ecuador have relied on a combination of smear microscopy, culture, and GeneXpert assay, with the choice of method varying based on each laboratory’s resources and availability. Since 2024, the national strategy for TB diagnosis has aimed to transition exclusively to a GeneXpert-based model, supported by the Ministry of Public Health’s acquisition of multiple GeneXpert devices to equip laboratories across the country. Although smear microscopy is quick and inexpensive, its low sensitivity and inability to differentiate between mycobacterial species limit its effectiveness.^10^ Culture, though highly sensitive and cost-effective, requires 6–8 weeks to yield results.^11^ In contrast, GeneXpert offers a rapid and reliable diagnostic solution. However, reliance on a single supplier for test components and the associated costs remain significant challenges, particularly for low- and middle-income countries.

Alternatively, commercial real-time PCR kits for MTBC detection, which rely on conventional DNA extraction from clinical specimens such as sputum, offer a promising diagnostic approach. Polymerase chain reaction-based detection of pathogen DNA is generally faster and more sensitive than traditional methods like microscopy or culture. Moreover, protocols involving DNA extraction either with column- or bead-based commercial kits or automated systems, followed by real-time PCR, are often more cost-effective than automated systems like GeneXpert. Both commercial PCR kits and in-house PCR protocols can be used, with the latter offering further cost reductions.

Our study highlights two key advantages of using the VIASURE or ANYPLEX kits for TB diagnosis: 1) significant time savings, as results can be delivered on the same day as sample collection, and 2) superior sensitivity not only compared with smear microscopy and culture but also, unexpectedly, to GeneXpert. However, a limitation of PCR diagnostics is the requirement for trained personnel proficient in molecular biology techniques, along with slightly longer processing times compared with GeneXpert.^12^ Moreover, a limitation of the current study is that specificity was not analyzed against a health or other disease control group.

In conclusion, the use of conventional DNA extraction from clinical samples, followed by real-time PCR with a commercial kit, demonstrated significantly higher MTBC-positivity rates in our study population, indicating superior sensitivity for TB case detection. Given the persistent burden of tuberculosis, especially in low- and middle-income countries, these results highlight the urgent need to revisit and update diagnostic guidelines. The WHO should give higher priority to the integration of molecular diagnostic techniques, particularly PCR-based methods, into routine practice. Such a shift would enhance early detection and treatment initiation, ultimately improving TB control efforts and reducing mortality in resource-limited settings. These improvements would support countries’ efforts to achieve the “End TB” goals by 2035.
